# Optimizing the synthesis of yeast Beta-glucan via response surface methodology for nanotechnology application

**DOI:** 10.1186/s12866-023-02845-6

**Published:** 2023-04-20

**Authors:** Alshimaa A. Atta-Allah, Rania F. Ahmed, Azza A. M. Shahin, Enas A. Hassan, Heba Abd-Alla El-Bialy, Mohie Z. El-Fouly

**Affiliations:** 1 Radiation Microbiology Department, National Center for Radiation Research and Technology, Egyptian Atomic Energy Authority, Cairo, Egypt; 2 Agricultural Microbiology Department, Faculty of Agriculture, Ain Shams University, Cairo, Egypt

**Keywords:** β-glucan, *Kluyveromyces lactis*, *Meyerozyma guilliermondii*, Agro-industrial wastes, Plackett–Burman, Response surface methodology, β-glucan nanoparticles (βGN)

## Abstract

**Background:**

The production of biopolymers from waste resources is a growing trend, especially in high-population countries like Egypt. Beta-glucan (β-glucan) belongs to natural polysaccharides that are derived from plant and microbial origins. In this study, following increasing demands for β-glucan owing to its bioactive properties, a statistical model to enhance microbial β-glucan production was evaluated for its usefulness to the food and pharmaceutical industries. In addition, a trial to convert β-glucan polymer to nanostructure form was done to increase its bioactivity.

**Results:**

Ingredients of low-cost media based on agro-industrial wastes were described using Plackett–Burman and central composite design of response surface methodology for optimizing yeast β-glucan. Minerals and vitamin concentrations significantly influenced β-glucan yield for *Kluyveromyces lactis* and nitrogen and phosphate sources for *Meyerozyma guilliermondii*. The maximum predicted yields of β-glucan recovered from *K. lactis* and *M. guilliermondii* after optimizing the medium ingredients were 407 and 1188 mg/100 ml; respectively. For the first time, yeast β-glucan nanoparticles (βGN) were synthesized from the β-glucan polymer using N-dimethylformamide as a stabilizer and characterized using UV–vis spectroscopy, transmission electron microscope (TEM), dynamic light scattering (DLS) and Fourier transform infrared spectroscopy (FT-IR). The average size of βGN was about 300 nm as determined by DLS. The quantitative variation of functional groups between β-glucan polymer and βGN was evaluated by FT-IR for explaining the difference in their biological activity against Normal Homo sapiens-Hela contaminant and Hepatic cancer cell lines.

**Conclusions:**

Enriching the low-cost media based on agro-industrial wastes with nutritional ingredients improves the yield of yeast β-glucan. The present study succeeds to form β-glucan nanoparticles by a simple method.

**Supplementary Information:**

The online version contains supplementary material available at 10.1186/s12866-023-02845-6.

## Background

The yeast cell wall is a dynamic organelle that acts as an interface between the cell components and the surrounding environment [1]. It consists of two layers with distinctly different chemical structures. The outer layer is composed mainly of high electron density mannoproteins while the internal layer is less dense and formed from polysaccharide polymer namely Beta-glucan (β-glucan). It contains a linear backbone of (1,3)-linked D-glucose molecules with (1–6) side chains of various lengths [2]. β-glucan displays a wide range of health effects including antimicrobial, antitumor, antioxidant, anti-inflammatory, and immunomodulatory agent, as well as a regulator for blood cholesterol and glycemia [3]. The members of this family are classified as ‘non-self’ polysaccharides and are considered foreign antigens upon injection into the human body [4]. They can bind to specific cell surface receptors of the host cells; macrophages, dendritic cells, T-Lymphocytes, and granulated neutrophils [5]. They can stimulate the cells of the specific immune system including B and T cells. Alternatively, they also activate peripheral immune cells, cytotoxic activity, phagocytic activity, reactive oxygen species, and cytokine secretion as non-specific responses [6]. The protective action of the esterified form of β-glucan against individual or mixed toxins including ochratoxin A, aflatoxin B1, and T-2 mycotoxicosis has been documented [7]. Recently, it has been described that positively charged polymers such as β-glucan for boosting type 1 interferon response to RNA viruses including influenza and coronavirus [8]. In addition, β-glucan would have great potential in the treatment of fungal co-infections associated with COVID-19 [9].

The β-glucans family is also considered dietary fiber that modulates the intestinal microbiota to a healthier state by increasing the growth of beneficial microflora and inhibiting the growth of gastrointestinal pathogens [10]. They are frequently used in the food industry not only as non-caloric dietary fibers but also for improving the physical properties of food products such as water-retaining agents, emulsifying stabilizers, thickening agents, or fat and oil-binding agents [11]. Moreover, yeast β-glucans have been used as edible films and food packaging materials [12].

In the pharmaceuticals industry, β-glucan plays a potential role in drug delivery owing to multiple aldehydes and hydroxyl groups characteristic to its structure that may be easily modified to form a hollow central cavity suitable for transport and encapsulation of pharmaceutical ingredients [13]. The mechanism of drug delivery of β-glucan is based on chiral interactions not electrostatic/hydrophobic interactions that are conventionally used in drug delivery systems [14].

With unlimited applications of β-glucan, yeast candidates deserve marked attention as most of them are recognized as safe [15], have a high propagation rate, and possibly re-modifying the chemical composition of the cell wall and consequently the content of β-glucan by controlling the growth phase, cultivation conditions, and availability of carbon and nitrogen sources [16].

According to the annual report of the Central Agency for Public Mobilization and Statistics of Egypt, 75 million tons of agricultural waste are produced annually [17]. They are utilized in the manufacturing of non-traditional fodder and organic fertilizers and high-value products such as biogas, liquid fuels, paper pulp, and compressed wood panels [18–20]. Using these resources rich in carbon and nitrogen nutrients for the production of biomolecules such as yeast β-glucans is an economically efficient process with eco-friendly aspects.

Considering all these aspects, the present study was carried out to modulate β-glucan recovered from *Meyerozyma guilliermondii* and *Kluyveromyces lactis* upon growing them on fruit and vegetable wastes (FVWs) and whey; a byproduct of cheese manufacture. The study also optimized the production process by using a response surface methodology. In addition, β-glucan nanoparticles (βGN) were synthesized and characterized via standard methods. The cytotoxicity of βGN on two cell lines was determined for medical applications.

## Results

### Optimizing β-glucan biosynthesis in non-conventional media

**Fig. 1:**
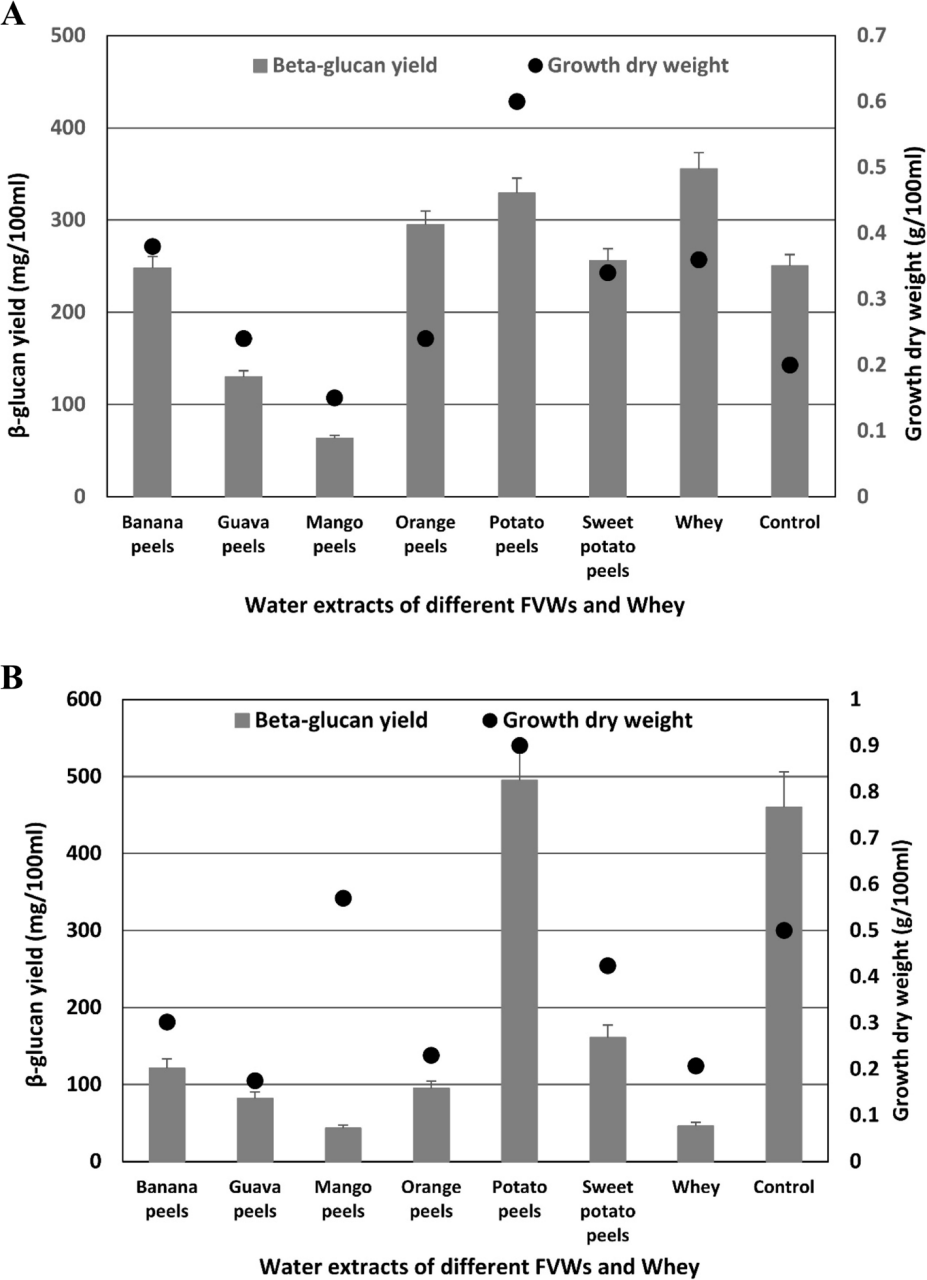
**A** Pattern of β-glucan yield and dry weight of *Kluyveromyces lactis* upon grown on different water extracts of fruit and vegetable wastes (FVWs) and whey. **B** Pattern of β-glucan yield and dry weight of *Meyerozyma guilliermondii* upon grown on different water extracts of fruit and vegetable wastes (FVWs) and whey

This study tested six fruit and vegetable wastes (FVWs) and whey for supporting β-glucan biosynthesis by *Meyerozyma guilliermondii* and *Kluyveromyces lactis*. The total carbohydrate contents of the water extract of FVWs and whey were determined as described in the methods section to prepare an equimolar concentration of these extracts. Figure 1A and B revealed that whey supports the maximum β-glucan biosynthesis by *K. lactis* (355.5 mg/100 ml) followed by water extract of potato peels (329 mg/100 ml). Regarding *M.* *guilliermondii*, the water extract of potato peels has the maximum impact on enhancing β-glucan biosynthesis (495 mg/100 ml) whereas whey failed to support a comparable quantity. There is no obvious relation between the yeast growth and the β-glucan yield as the growth of *K. lactis* on water extract of potato peels is higher than that on whey but the opposite is true for β-glucan yield.

Plackelt-Burman experimental design is based on the balanced incomplete blocks that use the least experimental runs to study the main effect of factors and fast screen the most significant factors among numerous variables. Twenty runs were selected to evaluate the impact of five factors; nitrogen, phosphate, and sulfate sources as well as minerals and vitamins on β-glucan biosynthesis. The results of fractional factorial design are shown in Tables 1 and 2. The β-glucan biosynthesis was markedly varied in a range from 83.4 to 332.5 mg/100 ml for *K. lactis* and from 190.1 to 662.6 mg/100 ml for *M.* *guilliermondii*. Among the screened factors for β-glucan biosynthesis by *K. lactis*, vitamin concentration has the highest impact on the β-glucan biosynthesis as given by the highest linear coefficient (23.9), followed by the mineral concentration (23.2). Similarly, phosphate concentration had the greatest effect on β-glucan biosynthesis by *M.* *guilliermondii* as indicated by the highest value of linear coefficient (57.6), followed by nitrogen source (50.3) but in an opposite sign. This means they are negatively related to each other. i.e. increasing the level of one factor and decreasing the level of another factor to maximize the β-glucan biosynthesis. The nitrogen, phosphate, and sulfate concentrations have a non-significant effect on β-glucan biosynthesis by *K. lactis* at a 90% of probability level as shown in Pareto charts of the Blackett-Burman design (Fig. S1). This can be ascribed to the richness of fresh whey with these nutrients especially the nitrogen source that contributes 8.28% as determined (Data not shown). On the other hand, minerals, and vitamins were non-significant factors for β-glucan biosynthesis by *M.* *guilliermondii* at α = 0.15 (Fig. S2) when that yeast was grown on water extract of potato peel.

**Table 1:** ANOVA, regression analysis, and significance of the Plackett–Burman experimental design to estimate the influential variables for β-glucan biosynthesis by *Kluyveromyces lactis*

Run Order			Nitrogen conc. (gl^−1^)	Sulfates conc. (gl^−1^)	Phosphates conc. (gl^−1^)	Minerals conc. (ml/l)	Vitamins conc. (gl^−1^)	β-glucan Conc. (mg/100 ml)
1			0.9 (-1)	1.0 (+ 1)	0.6 (-1)	15.0 (+ 1)	0.50 (+ 1)	285.8
2			9.0 (+ 1)	0.1 (-1)	0.6 (-1)	15.0 (+ 1)	0.50 (+ 1)	271.3
3			0.9 (-1)	0.1 (-1)	0.6 (-1)	1.5 (-1)	0.50 (+ 1)	210.7
4			9.0 (+ 1)	1.0 (+ 1)	0.6 (-1)	1.5 (-1)	0.05 (-1)	181.6
5			0.9 (-1)	1.0 (+ 1)	0.6 (-1)	15.0 (+ 1)	0.05 (-1)	218.3
6			9.0 (+ 1)	1.0 (+ 1)	0.6 (-1)	15.0 (+ 1)	0.50 (+ 1)	332.5
7			9.0 (+ 1)	0.1 (-1)	0.6 (-1)	1.5 (-1)	0.05 (-1)	146.4
8			0.9 (-1)	0.1 (-1)	0.6 (-1)	15.0 (+ 1)	0.05 (-1)	083.4
9			0.9 (-1)	1.0 (+ 1)	6.0 (+ 1)	1.5 (-1)	0.50 (+ 1)	199.9
10			9.0 (+ 1)	1.0 (+ 1)	6.0 (+ 1)	1.5 (-1)	0.05 (-1)	161.5
11			0.9 (-1)	1.0 (+ 1)	6.0 (+ 1)	15.0 (+ 1)	0.50 (+ 1)	252.5
12			0.9 (-1)	0.1 (-1)	0.6 (-1)	1.5 (-1)	0.05 (-1)	202.3
13			0.9 (-1)	1.0 (+ 1)	6.0 (+ 1)	1.5 (-1)	0.05 (-1)	261.3
14			9.0 (+ 1)	0.1 (-1)	6.0 (+ 1)	15.0 (+ 1)	0.05 (-1)	241.9
15			9.0 (+ 1)	1.0 (+ 1)	6.0 (+ 1)	15.0 (+ 1)	0.05 (-1)	245.1
16			0.9 (-1)	0.1 (-1)	6.0 (+ 1)	1.5 (-1)	0.50 (+ 1)	128.9
17			9.0 (+ 1)	0.1 (-1)	6.0 (+ 1)	1.5 (-1)	0.50 (+ 1)	278.9
18			9.0 (+ 1)	1.0 (+ 1)	0.6 (-1)	1.5 (-1)	0.50 (+ 1)	261.5
19			0.9 (-1)	0.1 (-1)	6.0 (+ 1)	15.0 (+ 1)	0.05 (-1)	284.5
20			9.0 (+ 1)	0.1 (-1)	6.0 (+ 1)	15.0 (+ 1)	0.50 (+ 1)	281.1
**Model Summary**								
**Source**	**DF**	**Adj. SS**	**Adj. MS**	***F*-Value**	***P*-Value**	**Effect**	**Coefficient**	***T*-Value**
**Model**	5	30540	6108	1.97	0.145			
**Linear**	5	30540	6108	1.97	0.145			
**Constant**							226.5	18.21
**Nitrogen**	1	3754	3754	1.21	0.289	27.4	13.7	1.10
**Sulfates**	1	3667	3667	1.19	0.295	27.1	13.5	1.09
**Phosphates**	1	1012	1012	0.33	0.576	14.2	7.1	0.57
**Minerals**	1	10728	10728	3.47	0.084	46.3	23.2	1.86
**Vitamins**	1	11380	11380	3.68	0.076	47.7	23.9	1.92
**Error**	14	43305	3093					
**Total**	19	73845

**Table 2:** ANOVA, regression analysis, and significance of the Plackett–Burman experimental design to estimate the influential variables for β-glucan biosynthesis by *Meyerozyma guilliermondii*

Run Order			Nitrogen conc. (gl^−1^)	Sulfates conc. (gl^−1^)	Phosphates conc. (gl^−1^)	Minerals conc. (ml/l)	Vitamins conc. (gl^−1^)	β-glucan Conc. (mg/100 ml)
1			0.7 (-1)	1.0 (+ 1)	0.6 (-1)	15.0 (+ 1)	0.50 (+ 1)	662.6
2			0.7 (-1)	1.0 (+ 1)	6.0 (+ 1)	15.0 (+ 1)	0.50 (+ 1)	549.8
3			0.7 (-1)	1.0 (+ 1)	6.0 (+ 1)	1.5 (-1)	0.50 (+ 1)	431.0
4			7.0 (+ 1)	1.0 (+ 1)	0.6 (-1)	1.5 (-1)	0.50 (+ 1)	308.8
5			7.0 (+ 1)	0.1 (-1)	6.0 (+ 1)	15.0 (+ 1)	0.50 (+ 1)	357.7
6			7.0 (+ 1)	1.0 (+ 1)	0.6 (-1)	1.5 (-1)	0.05 (-1)	211.9
7			7.0 (+ 1)	0.1 (-1)	6.0 (+ 1)	15.0 (+ 1)	0.05 (-1)	411.2
8			0.7 (-1)	0.1 (-1)	0.6 (-1)	1.5 (-1)	0.05 (-1)	260.7
9			0.7 (-1)	0.1 (-1)	0.6 (-1)	1.5 (-1)	0.50 (+ 1)	280.5
10			7.0 (+ 1)	1.0 (+ 1)	6.0 (+ 1)	15.0 (+ 1)	0.05 (-1)	299.6
11			0.7 (-1)	0.1 (-1)	0.6 (-1)	15.0 (+ 1)	0.05 (-1)	212.3
12			0.7 (-1)	1.0 (+ 1)	0.6 (-1)	15.0 (+ 1)	0.05 (-1)	623.7
13			7.0 (+ 1)	1.0 (+ 1)	6.0 (+ 1)	1.5 (-1)	0.05 (-1)	523.4
14			0.7 (-1)	0.1 (-1)	6.0 (+ 1)	1.5 (-1)	0.50 (+ 1)	572.2
15			7.0 (+ 1)	0.1 (-1)	0.6 (-1)	15.0 (+ 1)	0.50 (+ 1)	442.2
16			0.7 (-1)	0.1 (-1)	6.0 (+ 1)	15.0 (+ 1)	0.05 (-1)	433.6
17			7.0 (+ 1)	0.1 (-1)	6.0 (+ 1)	1.5 (-1)	0.50 (+ 1)	520.1
18			7.0 (+ 1)	1.0 (+ 1)	0.6 (-1)	15.0 (+ 1)	0.50 (+ 1)	190.1
19			7.0 (+ 1)	0.1 (-1)	0.6 (-1)	1.5 (-1)	0.05 (-1)	312.2
20			0.7 (-1)	1.0 (+ 1)	6.0 (+ 1)	1.5 (-1)	0.05 (-1)	557.7
**Model Summary**								
**Source**	**DF**	**Adj. SS**	**Adj. MS**	***F*-Value**	***P*-Value**	**Effect**	**Coefficient**	***T*-Value**
**Model**	5	145489	29098	1.57	0.231			
**Linear**	5	145489	29098	1.57	0.231			
**Constant**							408.1	13.42
**Nitrogen**	1	50692	50692	2.74	0.120	-100.7	-50.3	-1.66
**Sulfates**	1	15451	15451	0.83	0.376	55.6	27.8	0.91
**Phosphates**	1	66275	66275	3.58	0.079	115.1	57.6	1.89
**Minerals**	1	2087	2087	0.11	0.742	20.4	10.2	0.34
**Vitamins**	1	10984	10984	0.59	0.454	46.9	23.4	0.77
**Error**	14	259072	18505					
**Total**	19	404561						

Twenty-six experiments were designed according to the central composite design of the response surface model. Then, the multiple regression analyses on the experimental data were carried out. Results revealed the model had a very low P-value and a high F-value of more than 50 (Tables 3 and 4) which indicates that the significance of the regression model is very high and a very small chance of the model could be due to noise. It was observed that the quadratic terms ${X}_{1}^{2} \mathrm{and} {X}_{2}^{2}$ are very significant. i.e. the sum of squares of vitamins and minerals for *K. lactis* and nitrogen and phosphate sources for *M.* *guilliermondii* are very significant; F value > 100. The lack of fit for the sum of squares is an important aspect of ANOVA which exhibits the functional relationship between experimental factors and response variables. A non-significant lack of fit values (0.244 for *M.* *guilliermondii* and 0.80 for *K. lactis*) depicts the insignificant proportionality of lack of fit to pure error and the experimental data obtained fitted well with the model.

**Table 3:** ANOVA, regression analysis, and significance of the response surface methodology to optimize β-glucan biosynthesis by *Kluyveromyces lactis*

Run Order		Minerals conc. (ml/l)		Vitamins conc. (gl^−1^)		β-glucan Conc. (mg/100 ml)	
1		1.50 (-1)		0.500 (+ 1)		324.5 ± 4.4	
2		1.50 (-1)		0.050 (-1)		300.0 ± 5.0	
3		8.25 (0)		0.275 (0)		403.0 ± 3.1	
4		8.25 (0)		0.275 (0)		403.0 ± 3.1	
5		8.25 (0)		0.275 (0)		431.0 ± 24.9	
6		8.25 (0)		0.005 (-1.2)		322.1 ± 9.0	
7		8.25 (0)		0.275 (0)		403.0 ± 4.4	
8		8.25 (0)		0.545 (+ 1.2)		293.3 ± 29.4	
9		1.50 (-1)		0.050 (-1)		290.5 ± 5.0	
10		1.50 (-1)		0.500 (+ 1)		333.3 ± 4.4	
11		8.25 (0)		0.275 (0)		403.0 ± 4.4	
12		8.25 (0)		0.275 (0)		403.0 ± 4.4	
13		8.25 (0)		0.275 (0)		403.0 ± 4.4	
14		15.00 (+ 1)		0.050 (-1)		372.0 ± 6.0	
15		0.15 (-1.2)		0.275 (0)		345.5 ± 2.8	
16		8.25 (0)		0.275 (0)		403.0 ± 4.4	
17		0.15 (-1.2)		0.275 (0)		340.0 ± 2.8	
18		8.25 (0)		0.275 (0)		393.3 ± 12.8	
19		16.35 (+ 1.2)		0.275 (0)		375.3 ± 5.7	
20		8.25 (0)		0.005 (-1.2)		340.0 ± 9.0	
21		15.00 (+ 1)		0.050 (-1)		360.0 ± 6.0	
22		15.00 (+ 1)		0.500 (+ 1)		289.1 ± 6.5	
23		16.35 (+ 1.2)		0.275 (0)		364.0 ± 5.7	
24		8.25 (0)		0.275 (0)		416.0 ± 9.9	
25		8.25 (0)		0.545 (+ 1.2)		352.0 ± 29.4	
26		15.00 (+ 1)		0.500 (+ 1)		302.0 ± 6.5	
**Model Summary**							
**Source**	**DF**	**Adj. SS**	**Adj. MS**	***F*-Value**	***P*-Value**	**Coefficient**	***T*-Value**
**Model**	5	45,039.0	9007.8	53.99			
**Linear**	2	2050.3	1025.2	6.14	0.008		
**Nitrogen Conc**	1	1411.4	1411.4	8.46	0.009	12.15	2.91
**Phosphate Conc**	1	638.9	638.9	3.83	0.064	-8.18	-1.96
**Square**	2	37,570.3	18,785.2	112.59			
Nitrogen*Nitrogen	1	9592.5	9592.5	57.50	< 0.001	-47.13	-7.58
Phosphate* Phosphate	1	25,260.0	25,260.0	151.40	< 0.001	-76.48	-12.30
**2-Way Interaction**	1	5418.4	5418.4	32.48			
Nitrogen*Phosphate	1	5418.4	5418.4	32.48	< 0.001	-37.48	-5.70
**Error**	20	3336.8	166.8				
**Lack-of-Fit**	3	186.6	62.2	0.34	0.800		
**Pure Error**	17	3150.2	185.3				
**Total**	25	48375.8

**Table 4:** ANOVA, regression analysis, and significance of the response surface methodology to optimize β-glucan biosynthesis by *Meyerozyma guilliermondii*

Run Order		Nitrogen source conc. (gl^−1^)		Phosphate source conc. (gl^−1^)		β-glucan Conc. (mg/100 ml)	
1		7.00 (+ 1)		6.00 (+ 1)		657.1 ± 52.7	
2		3.85 (0)		3.30 (0)		514.0 ± 5.1	
3		0.70 (-1)		6.00 (+ 1)		1050.1 ± 54.1	
4		0.70 (-1)		0.60 (-1)		835.0 ± 30.9	
5		3.85 (0)		3.30 (0)		502.0 ± 17.1	
6		0.07 (-1.2)		3.30 (0)		770.6 ± 0.2	
7		7.00 (+ 1)		6.00 (+ 1)		762.3 ± 52.7	
8		0.70 (-1)		0.60 (-1)		773.2 ± 30.9	
9		3.85 (0)		3.30 (0)		523.4 ± 4.3	
10		3.85 (0)		3.30 (0)		533.0 ± 13.9	
11		3.85 (0)		3.30 (0)		528.7 ± 9.6	
12		3.85 (0)		3.30 (0)		502.0 ± 17.1	
13		3.85 (0)		3.30 (0)		514.0 ± 5.1	
14		3.85 (0)		3.30 (0)		523.4 ± 4.3	
15		3.85 (0)		3.30 (0)		533.0 ± 13.9	
16		3.85 (0)		0.06 (-1.2)		723.4 ± 40.6	
17		3.85 (0)		6.54 (+ 1.2)		827.0 ± 9.0	
18		3.85 (0)		0.06 (-1.2)		642.2 ± 40.6	
19		0.70 (-1)		6.00 (+ 1)		942.0 ± 54.1	
20		7.00 (+ 1)		0.60 (-1)		778.7 ± 56.2	
21		7.00 (+ 1)		0.60 (-1)		891.0 ± 56.2	
22		3.85 (0)		6.54 (+ 1.2)		809.0 ± 9.0	
23		7.63 (+ 1.2)		3.30 (0)		746.0 ± 0.5	
24		7.63 (+ 1.2)		3.30 (0)		747.0 ± 0.5	
25		3.85 (0)		3.30 (0)		517.5 ± 1.6	
26		0.07 (-1.2)		3.30 (0)		770.2 ± 0.2	
**Model Summary**							
**Source**	**DF**	**Adj. SS**	**Adj. MS**	***F*-Value**	***P*-Value**	**Coefficient**	***T*-Value**
**Model**	5	572432	114486	50.99			
**Linear**	2	38590	19295	8.59	0.002		
**Nitrogen Conc**	1	15956	15956	7.11	0.015	-40.6	-2.67
**Phosphate Conc**	1	22635	22635	10.08	0.005	48.7	3.18
**Square**	2	466461	233230	103.88			
Nitrogen*Nitrogen	1	222414	222414	99.06	< 0.001	226.9	9.95
Phosphate* Phosphate	1	206915	206915	92.16	< 0.001	218.9	9.60
**2-Way Interaction**	1	67381	67381	30.01			
Nitrogen*Phosphate	1	67381	67381	30.01	< 0.001	-132.2	-5.48
**Error**	20	44903	2245				
**Lack-of-Fit**	3	9517	3172	1.52	0.244		
**Pure Error**	17	35386	2082				
**Total**	25	617335					

By analysis of the variance for data that were represented in Tables 3 and 4, the adjusted R^2^ value for *K. lactis* equals 0.9138 which is close to *R*^2^ = 0.9310, and the adjusted R^2^ value for *M.* *guilliermondii* equals 0.9091 which is also close to *R*^2^ = 0.9273. The discrepancy between the above values is less than 0.02, this means the model can reliably predict a response model.

The predictive model for β-glucan biosynthesis in *K. lactis* was as follows:

$${\mathbf Y}_{\mathbf1}=234.5+18.07\ast{\mathbf X}_{\mathbf1}+688.1\ast{\mathbf X}_{\mathbf2}-0.7183\ast\mathbf X_{\mathbf1}^{\mathbf2}-1049.1\ast\mathbf X_{\mathbf2}^{\mathbf2}-17.14\ast{\mathbf X}_{\mathbf1}\ast{\mathbf X}_{\mathbf2}$$

Where X_1_ is the mineral concentration, X_2_ is the vitamin concentration as yeast extract and Y_1_ (β-glucan yield) is significant on the level of α = 0.1. Both factors showed a significant negative quadratic effect on β-glucan yield indicating that β-glucan biosynthesis was elevated with increasing the levels of these factors but showed a significant decrease as these factors increased to higher levels.

Similarly, the predictive model for β-glucan yield in *M.* *guilliermondii* was as follows,

$${\mathbf Y}_{\mathbf1}=838.8-81.1\ast{\mathbf X}_{\mathbf1}-97.5\ast{\mathbf X}_{\mathbf2}+20.85\ast\mathbf X_{\mathbf1}^{\mathbf2}+15.88\ast\mathbf X_{\mathbf2}^{\mathbf2}-10.79\ast{\mathbf X}_{\mathbf1}\ast{\mathbf X}_{\mathbf2}$$

Where X_1_ is a phosphate source concentration, X_2_ is a nitrogen source concentration and Y_1_ (β-glucan yield) is significant on the level of α = 0.1.

**Fig. 2:**
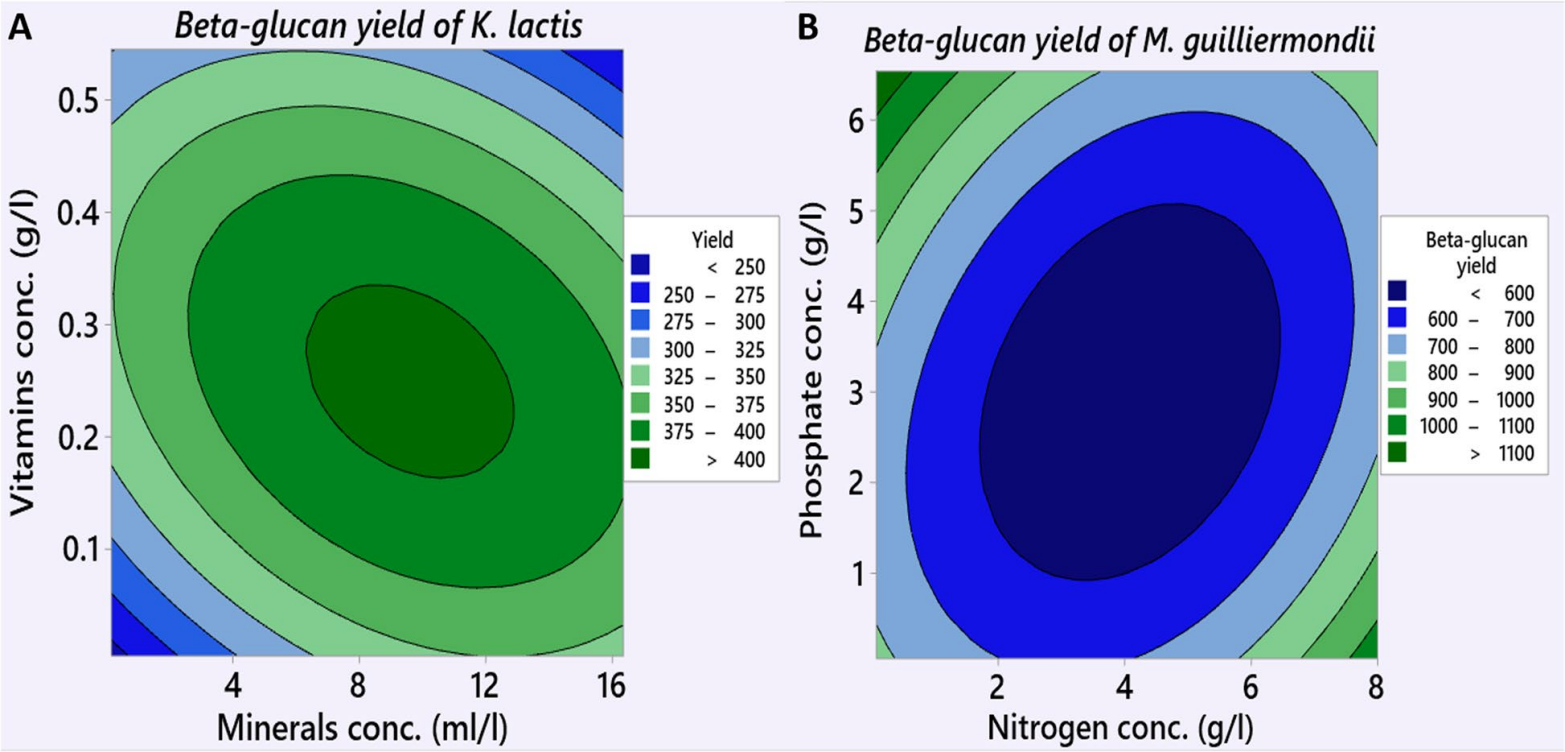
Contour plots of the most significant variables affecting β-glucan biosynthesis. **A** Minerals and vitamins concentration for *Kluyveromyces lactis*. **B** Nitrogen and Phosphate concentrations for *Meyerozyma guilliermondii*

Figure 2 (A and B) shows the contour plots of β-glucan yield for each pair of factors; either vitamins and minerals concentrations for *K. lactis* or nitrogen and phosphate concentrations for *M.* *guilliermondii*; respectively whereas the other factors (that are studied in screening Plackett–Burman design) were kept constant at their low level to reduce the production costs. The β-glucan yield of *K. lactis* increased (> 400 mg/100 ml) with elevated vitamins and mineral concentrations, in the ranges between 0.2–0.3 g and 8–11 ml per liter; respectively. Further increase in the level of these factors resulted in a decrease in β-glucan yield. Similarly, the β-glucan level of *M.* *guilliermondii* increased by elevating phosphate concentration to more than 5.5gl^−1^ and declining the nitrogen concentration to less than 1.0 gl^−1^.

**Fig. 3:**
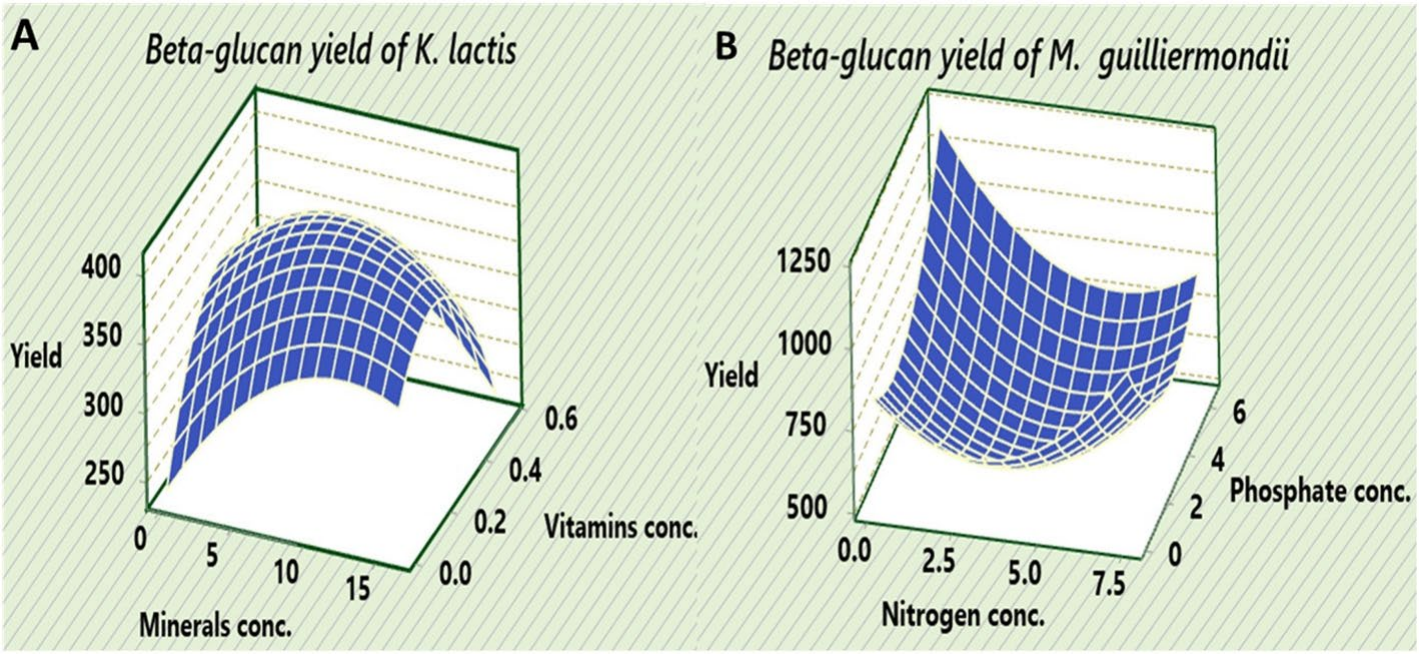
Surface plots of the most significant variables affecting β-glucan biosynthesis. **A** Minerals and vitamins concentration for *Kluyveromyces lactis*. **B** Nitrogen and Phosphate concentrations for *Meyerozyma guilliermondii*

The interaction between different variables (X_1_*X_2_) is shown in Fig. 3 (A and B) by surface plots. With increasing the mineral concentration from 10 ml to about 12 ml; the β-glucan yield of *K. lactis* drifted down and then slightly decreased when the mineral concentration was increased up to 15 ml. For vitamins added to the low-cost medium based on whey, the middle concentration (≃ 0.25 gl^−1^ of yeast extract) supports the maximum β-glucan biosynthesis. The interaction between the nitrogen source (X_1_) and the phosphate source (X_2_) on the β-glucan yield by *M.* *guilliermondii* showed a decrease in the nitrogen concentration to the minimum level in the model (0.07 gl^–1^) and the increase in phosphate concentration to the maximum value in the model (6.540 gl^–1^) had the highest impact on the β-glucan yield followed by run 19 which had a low nitrogen concentration; 0.7 gl^–1^ and a high phosphate conc.; 6.0 gl^–1^ (Table 4). The reverse relationship of these factors is also confirmed by the negative value of the interaction coefficient for nitrogen and phosphate, -132.2 (Table 4). Finally, the optimizer of the model predicted the β-glucan yields of *K. lactis* and *M. guilliermondii*, 407 and 1188 mg/100 ml; respectively when keeping the variables under investigation at optimal levels. Any change in the variables’ concentrations either by increasing or decreasing above the optimal values negatively affects β-glucan yields (Fig. S3). The optimum values of minerals and vitamins for *K. lactis* were 9.6409 ml and 0.2505 g^−1^; respectively whereas the optimum values of nitrogen and phosphate concentrations for *M. guilliermondii* were 0.07 and 6.54 gl^−1^; on the same order.

### Synthesis and characterization of β-glucan nanoparticles

**Fig. 4:**
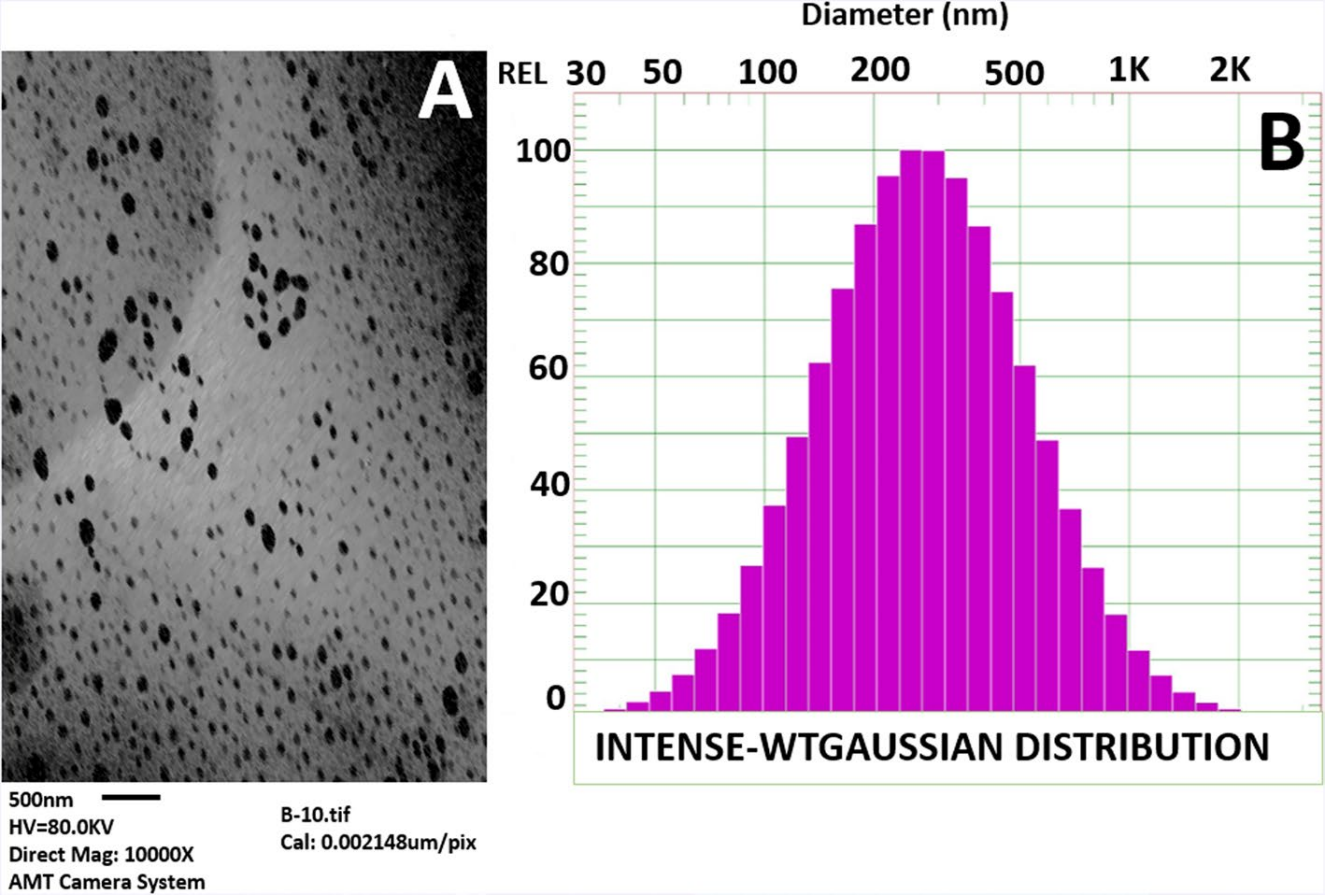
Characterization of the size of β-glucan nanoparticles using. **A** Transmission Electron Microscope (TEM). **B** Dynamic Light Scattering (DLS)

This work succeeds to synthesize β-glucan nanoparticles (βGN) using N-dimethylformamide as a new stabilizer that wasn’t used before in βGN formation. TEM micrograph of βGN showed spherical shape particles with smooth surfaces. The size of formed nanoparticles has a broad range; from smaller than 20 nm to larger than 90 nm as shown in Fig. 4A. UV–Vis spectra of βGN exhibited a broad peak (220-300 nm) resembling the successful formation of nanoparticles (Data not shown). The average size of βGN as determined by DLS is larger than that selected by TEM (More than 300 nm; Fig. 4B).

**Fig. 5:**
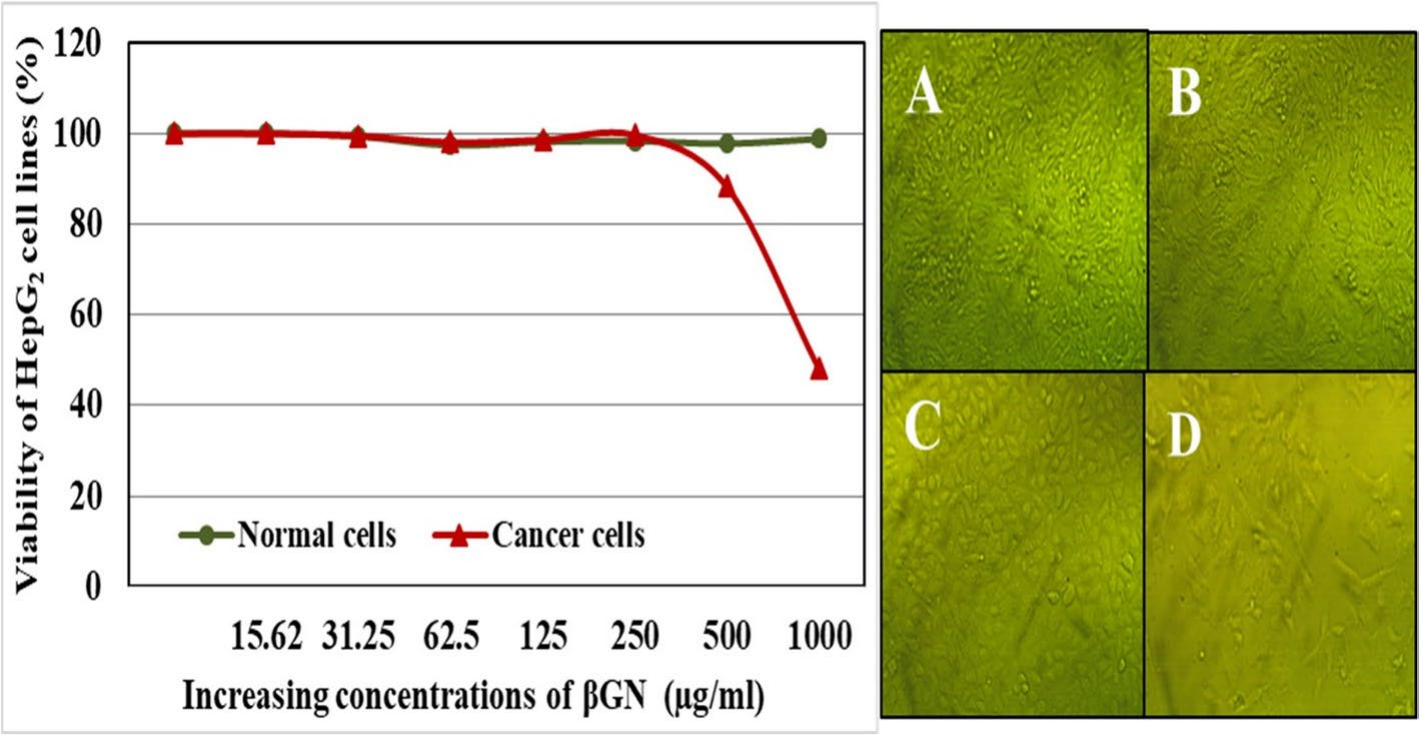
Cytotoxicity of different concentrations of β-glucan nanoparticles on HepG2 cell line. **A** HepG2-Normal cells treated with zero concentration of β-glucan nanoparticles. **B** HepG2-Normal cells treated with 1000 µg/ml of β-glucan nanoparticles. **C** HepG2-Cancer cells treated with zero concentration of β-glucan nanoparticles. **D** HepG2-Cancer cells treated with 1000 µg/ml of β-glucan nanoparticles

**Fig. 6:**
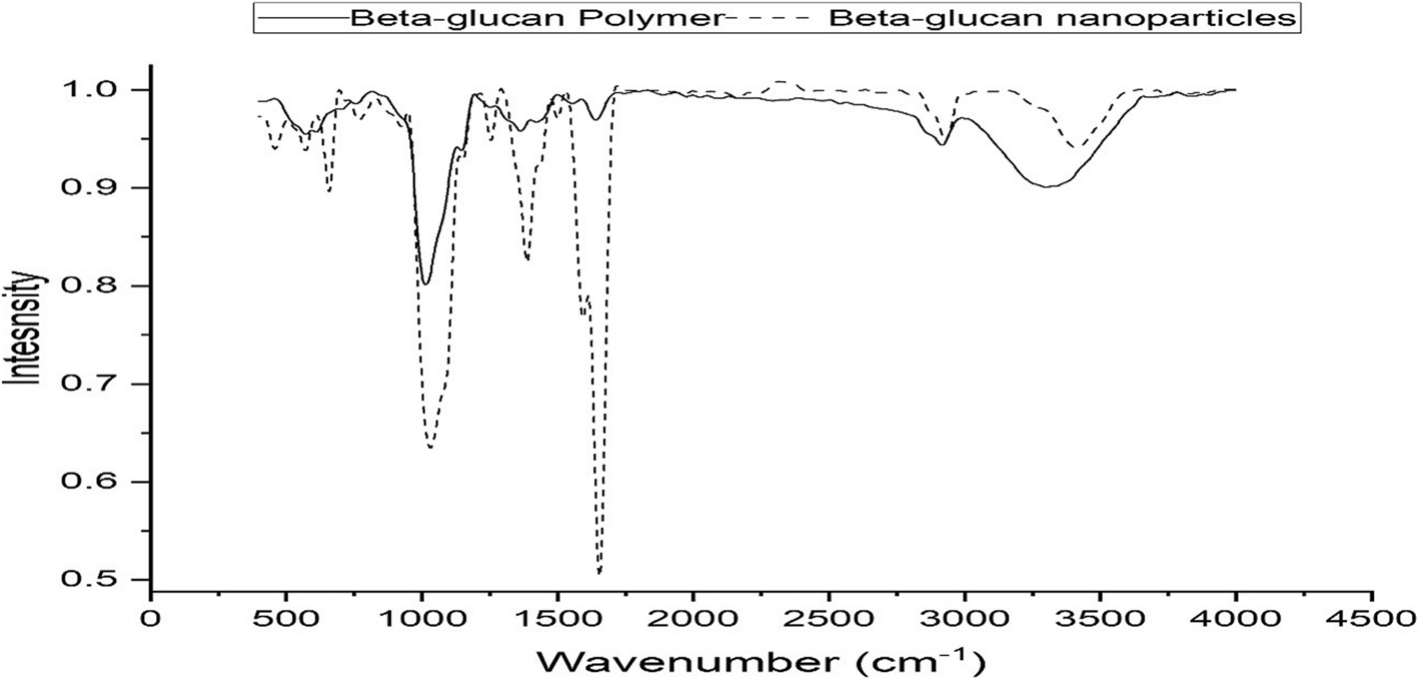
Fourier transform infrared spectroscopy (FT-IR) graphs showing functional groups present in β-glucan polymer and β-glucan nanoparticles

In the present study, the antitumor activity of βGN was evaluated using an MTT assay. Figure 5 showed a negative effect of βGN on the viability of Normal Homo sapiens-Hela contaminant and Hepatic cancer (HepG_2_) cell lines up to 250 µg/ml. The viability of HepG_2_ cell line declined gradually and reached to nearly 50% at 1000 µg/ml whereas no lethal effect was observed on the viability of normal cell lines. The cytotoxicity of βGN on the HepG_2_ cell line was lower than that observed by β-glucan polymer (Data not shown). To explain the decrease in the biological activity of βGN, the functional groups of βGN were studied in comparison with β-glucan polymer using FT-IR spectroscopy. Figure 6 illustrates a number of characteristic peaks at 571.76, 1013.67, 1640.59, 2916.58, and 3300.17 cm^−1^ for both β-glucan polymer and βGN but with a great difference in intensity.

## Discussion

With the increase in the biomass of fruit and vegetable wastes (FVWs) from various industries around the world, great efforts are improved to reduce the negative effects of these wastes on the environment, especially if solving the problem is accompanied by the development of value-added products. Microbial fermentation of FVWs has been shared in the global market of many biotechnology processes including alcohols, enzymes, organic and amino acids, and biopolymers production [21]. In the present study, two non-conventional media based on agro-industrial wastes; water extract of potato peels and whey were selected for enhancing β-glucan biosynthesis by *Meyerozyma guilliermondii* and *Kluyveromyces lactis*; respectively. Previous research suggested the β-glucan yield will be in line with the cell growth [22]. Utama and his collaborators correlated the highest microbial β-glucan yield to the carbohydrate content of the growth-supporting waste [23]. Alternatively, using a low-cost media based on potato juice and glycerol could alter the cell wall structure of *Candida utilis* ATCC 9950 by increasing the amounts of (1,3)/(1,6)-glucans [24].

The agro-industrial wastes mainly contain carbon sources that are vital for yeast growth but may not be sufficient for maximizing β-glucan biosynthesis. Thus, a set of statistical experiments were performed to complete the new media with ingredients that needed to be compatible with synthetic ones and also minimize the residual nutrients prior to the release of the effluent processing into the environment, this is important both from an economic point of view and for environmental concerns. The non-significant impact of vitamins and minerals on the β-glucan biosynthesis by *M.* *guilliermondii* could be attributed to the richness of potato peels in minerals and vitamins as previously revealed [25]. Similarly, the high content of protein and non-protein nitrogen in whey revealed the non-significance of nitrogen variable on the β-glucan biosynthesis by *K. lactis* [26].

The predictive model shows the negative linear effect of the nitrogen source (ammonium sulfate) and a positive quadratic effect of both nitrogen and phosphate concentration in the low-cost medium based on potato peel-water extract (As shown in Table 2). The medium based on water extract of the potato peels needs no additional nitrogen sources. This could be attributed to the richness of potato waste juice with nutrients like proteins (app. 2%) as previously found [27]. In addition, supplementing the low-cost medium based on potato peels with an additional nitrogen source may alter the C/N ratio and become an unsuitable ratio for fungal growth [28].

This research succeeded in synthesizing β-glucan nanoparticles (βGN) from the β-glucan polymer by a modified procedure [29] that was previously described using DEF as a stabilizer for nanoparticles. Han and collaborates synthesized trifluoroacetic acid-stabilized βGN with a minimum size; 250 nm [30], which was similar to the size of βGN attained by DLS in this study. This can be ascribed to the hydrodynamic state of βGN in DLS measurements whereas these nanoparticles are in the dry state during the TEM micrograph since βGN must be diluted by distilled water before DLS measurements. Beta-glucan is characterized by having a triple helix structure in an aqueous solution and can be separated into single strands in a strongly alkaline medium [31]. In the present study, the alkaline medium can be attained by NaOH used to generate βGN as described in the methods section. Dilution of β-glucan with distilled water at a rate of 1:20 or more converts the separated single strands of polysaccharide polymer into the triple helix structure which is known as renaturation. In this process, the fiber phase structure of the polymer is formed owing to Pi and hydrogen bonds as well as hydrophobic bonds. Thus, the combination of single and triple strands of β-glucan with different lengths in the mixture is the reason for the variation in the particle size of βGN [32].

Unlike, small molecular weight anticancer chemicals, polysaccharides are clinically used as successful antitumor agents with no toxic side effects [33]. The antitumor activity of β-glucan mainly depends on the inhibition of tumor cell proliferation by inducing apoptosis and activating the response of adaptive immune cells such as B-cells, CD-4 cells, and CD-8 cells [34]. The biological activity of β-glucan depends on the molecular weight, size, conformation, number of bonds, and solubility [2]. FT-IR spectroscopy is a powerful tool for analyzing the structural analysis of polysaccharides. This technique is sensitive to the anomeric configuration and binding position of in glucosidic linkage polysaccharides such as β-glucan [35]. A characteristic peak appeared at 571.76 cm^−1^ representing the skeletal vibration of glucose pyranose moiety [36]. The absorption peak at 1013.67 cm^−1^ revealed 1,3-linked D-glucose molecules forming the linear backbone of β-glucan. Several peaks can be easily recognized between 1200 and 1400 cm^−1^ which is mainly reflected to carbonyl linkage as previously described [37]. The band at 1640.59 cm^−1^ was related to the tightly bound water molecules in the β-glucan polymer. A similar finding was reported at 1645 cm^−1^ for the bending vibration of water molecules [38]. The absorption peak at 2916.58 cm^−1^ was assigned to the C-H stretching of the β-glucan polymer. The absorbance of aliphatic CH_x_ bands present in the range 2800–3000 cm^−1^ and the intensity of vibration of CH_3_ is greater than aliphatic CH_2_ or CH stretching vibration [39]. The band observed at 3300.17 cm^−1^ reflects the stretching of the hydroxyl group (O–H) that shares a hydrogen bond formation; the main cause of the helix structure of β-glucan [40]. As shown in Fig. 6, the FT-IR spectrum of βGN is not similar to that of β-glucan polymer. The intensity of glucose pyranose moiety, C_1,3_-linkage, and carbonyl linkage was increased due to the fragmentation of β-glucan polymer into nano-size. The dramatic increase in the water molecules regarding the βGN spectrum is attributed to the aqueous nature of the nanoparticle formation process. On the other hand, the intensity of the band represents hydroxyl group vibration (3300.17 cm^−1^) is lower in the βGN spectrum than that observed in the β-glucan polymer spectrum. The lower content of the hydroxyl group in βGN explains its lower activity against cancer cell lines in comparison to large-sized β-glucan polymer since the hydroxyl groups preserve the helix structure of β-glucan that favors its biological activity [41]. In addition, by decreasing the size of β-glucan to nano-size, a large number of non-reducing ends emerge that have a lower biological activity [42].

## Methods

### Microorganisms and agro-industrial wastes

Out of 49 yeasts, only two with a high β-glucan yield are selected in a preliminary experiment (Data not shown). The selected yeasts, *Kluyveromyces lactis,* and *Meyerozyma guilliermondii* were previously isolated from cottage cheese and fig fruit; respectively. They are identified at the species level by genetic tools. The sequences of the selected yeasts were compared with 18S rRNA gene sequencing in the Genbank database using the blast function. Then, the phylogenetic trees of both yeasts were drawn (Figs. S4 and S5). Their nucleotide sequences were accessed in the Genbank under accession numbers, OP967929 and OP967932; respectively.

Water extracts of six fruit and vegetable wastes (FVWs); banana peels, guava peels, and pulp, potato peels, orange peels, sweet potato peels, and mango peels (200 g/l) were individually steamed under pressure for 1 h. The extracts were filtered using four layers of cheesecloth to remove solid debris and kept at -4 °C until use. Fresh whey was prepared from full cream milk by the standard method as previously described [43]. The total concentration of carbohydrates present in FVWs and whey was determined by the phenol–sulfuric acid method [44]. The nitrogen content of whey was determined at the micro-analytical center, Agriculture Faculty, Ain-Shams University. The water extracts of different FVWs and whey were adjusted at equimolar concentrations of carbon source (20 g of reducing sugar/liter) for upcoming experiments.

### Precultures and cultivation conditions

Precultures were carried out in the yeast malt broth medium [45]. The inoculated cultures were incubated for 24 h at 20 °C. The growing cells were harvested in sterile saline (0.9%) under aseptic conditions and used as inoculum for fresh media (100 ml) based on water extracts of FVWs or whey supplemented with the following ingredients: KH_2_PO_4_ as phosphate source, MgSO_4_.7H_2_O as sulfate source, yeast extract as a vitamin-rich compound, and a mineral solution containing; ZnSO_4_.7H_2_O, 4.5 mg; CoCl_2_.6H_2_O, 0.3 mg; MnCl_2_.4H_2_O, 1 mg; FeSO_4_.7H_2_O, 3 mg; NaMoO_4_.2H_2_O, 0.4 mg; H_3_BO_3_, 1 mg; KI, 0.1 mg and EDTA, 0.1 mg per liter [46]. The equimolar concentrations of nitrogen source; 6.44 gl^−1^ sodium nitrate for *K. lactis* and 5 gl^−1^ ammonium sulfate for *M.* *guilliermondii*; were added to the low-cost media. The control medium contains 10.52 gl^−1^ lactose and 11.52 gl^−1^ maltose as the sole source of carbon for yeasts under investigation on the same order. Cultures of *K. lactis* and *M.* *guilliermondii* were incubated under shaking conditions (150 rpm) at 20 °C for 120 h and 72 h; respectively. The yeast cell mass was determined as dry cell weight.

### Recovery and analysis of β-glucan

At the end of the incubation periods, yeast cells were recovered from growing cultures. Half a gram of fresh yeast cells was suspended in 25 ml NaCl (3%) and autolyzed at 65°C for 40 h [47]. The autolyzed cells (mainly cell walls) were collected by centrifugation for 20 min. at 4500 rpm; the supernatant was discarded and the pellet was stored at 4 °C until use. Ten milliliters of 1.0 N NaOH were added to the yeast cell walls, and the mixture was heated at 95 °C for 2 h. The mixture was centrifuged and the pellet was repeatedly washed (Five times or more) with distilled water to reduce its alkalinity. Finally, purified β-glucan was quantitatively determined as a carbohydrate by the phenol–sulfuric acid method [44].

### Statistical optimization of β-glucan biosynthesis on low-cost cultivation media

Response surface methodology is a combination of statistical and mathematical methods used to select the best experimental conditions requiring the minimum number of experiments to achieve the maximum impact in the system of multiple factor [48, 49]. Nutrient components; nitrogen source, phosphate source, sulfate source, minerals, and vitamins that affect the performance of low-cost cultivation media based on whey for *K. lactis* and potato peels for *M. guilliermondii* were studied for enhancing β-glucan biosynthesis. In total, twenty experimental runs were conducted using a Plackett Burman design (Minitab V.18.1. software) with two levels encoded (+ 1, -1). The coefficients were evaluated using non-linear regression analysis to screen the significance of these independent variables.

To optimize the β-glucan biosynthesis by *K. lactis* and *M.* *guilliermondii,* a total of twenty-six experimental runs with two independent variables achieved the maximum yield of β-glucan in the previous experiment (minerals and vitamins for *K. lactis* & nitrogen and phosphate sources for *M.* *guilliermondii*) were designed with levels encoded (-1.2, -1.0, 0, + 1.0, + 1.2) by face central composite design. *P*-values and F-values were used to evaluate the overall significance of the regression model. The second order response function for two quantitative factors is given by the equation:

$$\mathrm Y=\;\beta_o+\;{\mathrm\beta}_1{\mathrm X}_1+\;{\mathrm\beta}_2{\mathrm X}_2+\;{\mathrm\beta}_{11}{({\mathrm X}_1)}^2+\;{\mathrm\beta}_{22}{({\mathrm X}_2)}^2+\;{\mathrm\beta}_{12}{\mathrm X}_1{\mathrm X}_2\;$$

where X_1_ and X_2_ represent the levels of the variables under investigation.

In addition, coefficient of determinations (R^2^) was used to estimate the quality of fitted regression model terms. Two dimensions (2-D) contour, three dimensions (3-D) response surface, and an optimizer chart were plotted to evaluate the response (β-glucan yield) under investigation.

### Biosynthesis of β-glucan nanoparticles (βGN)

One gram of lyophilized β-glucan was dissolved in 100 ml of sodium hydroxide (2%) and stirred at 90°C on the magnetic stirrer for 3 h [28]. Nanoparticles of β-glucan were precipitated by adding acetic acid (1%). The stabilization of the formed nanoparticles was performed using N-dimethylformamide (DEF).

### Characterization of β-glucan nanoparticles

The mixture containing nanoparticles was sonicated for 20 min., immediately dropped on a copper grid, and let dry for imaging with a JEOL 1230 high contrast transmission electron microscope to determine the size and morphology of formed nanoparticles. The absorption spectra of the βGN were measured in a wavelength range; 200–900 nm using a T60 UV–Vis spectrophotometer (China). The average particle size and size distribution of β-glucan nanoparticles were estimated using the dynamic light scattering technique (DLS) PSS-Nicomp 380-ZLS. The functional groups of βGN in comparison to β-glucan polymer were determined using a Bruker Vertex 70 FT-IR spectrophotometer in the range 4000–400 cm^−1^ at a resolution of 4 cm^−1^.

### Cytotoxicity of β-glucan polymer and β-glucan nanoparticles

The cytotoxicity of different concentrations of β-glucan nanoparticles (15.62–1000 µg/ml) was determined using Normal Homo sapiens-Hela contaminant and Hepatic cancer (HepG_2_) cell lines by (3-(4,5-dimethylthiazol-2-yl)-2,5-diphenyltetrazolium bromide (MTT). The MTT assay is based on the reduction of a yellow tetrazolium salt to purple formazan in the mitochondria of living cells; the reduction was quantified by measuring the absorbance at 560 nm [50].

## Supplementary Information

**Additional file 1: Figure (S1).** Plot of significance effects and Pareto chart of the variables influencing β-glucan biosynthesis by *Kluyveromyces lactis*. **Figure (S2).** Plot of significance effects and Pareto chart of the variables influencing β-glucan biosynthesis by *Meyerozyma guilliermondii*. **Figure (S3).** Response optimization plots of the most significant variables affecting β-glucan biosynthesis. A: Minerals and vitamins concentration for *Kluyveromyces lactis*. B: Nitrogen and Phosphate concentrations for *Meyerozyma guilliermondii*. **Figure S4.** Phylogenetic tree showing the relationship of the selected yeast *Kluyveromyces lactis*with other related fungal species relatives from Genbank based on their sequence homology of 18S rRNA. **Figure S5.** Phylogenetic tree showing the relationship of the selected yeast *Meyerozyma guilliermondii *with other related fungal species relatives from Genbank based on their sequence homology of 18S rRNA.

## Data Availability

The 18S rRNA sequence of *Kluyveromyces lactis* and *Meyerozyma guilliermondii* used in the present study are available in the GenBank repository with accession numbers; OP967929 and OP967932, respectively. Data not shown can be easily available from the corresponding author upon request.
