# Supplementary material for: Optimizing the synthesis of yeast Beta-glucan via response surface methodology for nanotechnology application
Source: BMC Microbiol. 2023 Apr 20;23:110. doi: 10.1186/s12866-023-02845-6 (PMC10116484; doi:10.1186/s12866-023-02845-6)
Supplement: Supplementary file 1 — Additional file 1: Figure (S1). Plot of significance effects and Pareto chart of the variables influencing β-glucan biosynthesis by Kluyveromyces lactis. Figure (S2). Plot of significance effects and Pareto chart of the variables influencing β-glucan biosynthesis by Meyerozyma guilliermondii. Figure (S3). Response optimization plots of the most significant variables affecting β-glucan biosynthesis. A: Minerals and vitamins concentration for Kluyveromyces lactis. B: Nitrogen and Phosphate concentrations for Meyerozyma guilliermondii. Figure S4. Phylogenetic tree showing the relationship of the selected yeast Kluyveromyces lactiswith other related fungal species relatives from Genbank based on their sequence homology of 18S rRNA. Figure S5. Phylogenetic tree showing the relationship of the selected yeast Meyerozyma guilliermondii with other related fungal species relatives from Genbank based on their sequence homology of 18S rRNA. [file 12866_2023_2845_MOESM1_ESM.docx]

**Supplementary materials**

**Optimizing the synthesis of yeast Beta-glucan via response surface methodology for nanotechnology application**

Alshimaa A. Atta-Allah^1^, Rania F. Ahmed^2^, Azza A. M. Shahin^1^, Enas A. Hassan^2^, Heba Abd-Alla El-Bialy^1*^ and Mohie Z. El-Fouly^1^

(1) Radiation Microbiology Department, National Center for Radiation Research and Technology, Egyptian Atomic Energy Authority, Cairo, Egypt

(2) Agricultural Microbiology Department, Faculty of Agriculture, Ain Shams University, Cairo, Egypt

**
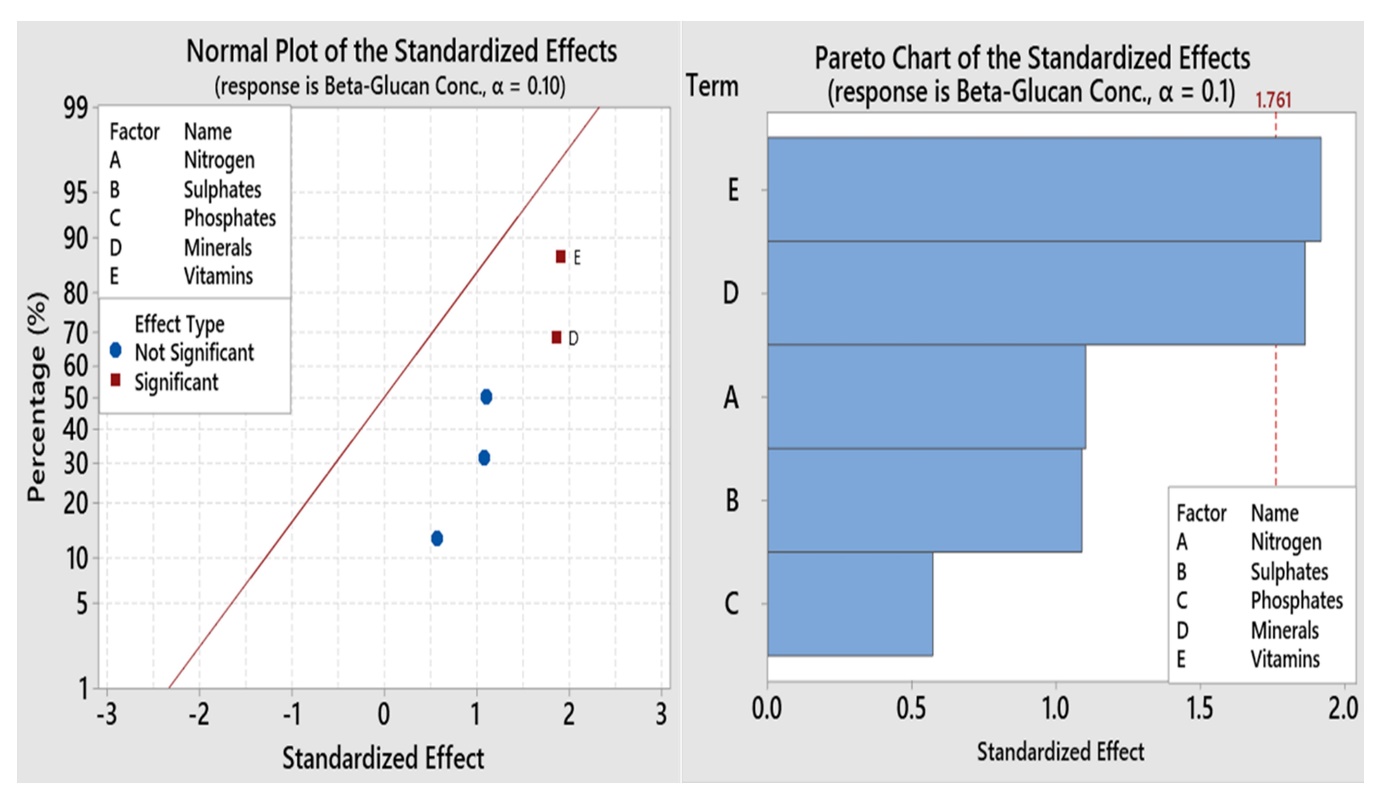
**

**Figure (S1): Plot of significance effects and Pareto chart of the variables influencing β-glucan biosynthesis by *Kluyveromyces lactis***

**
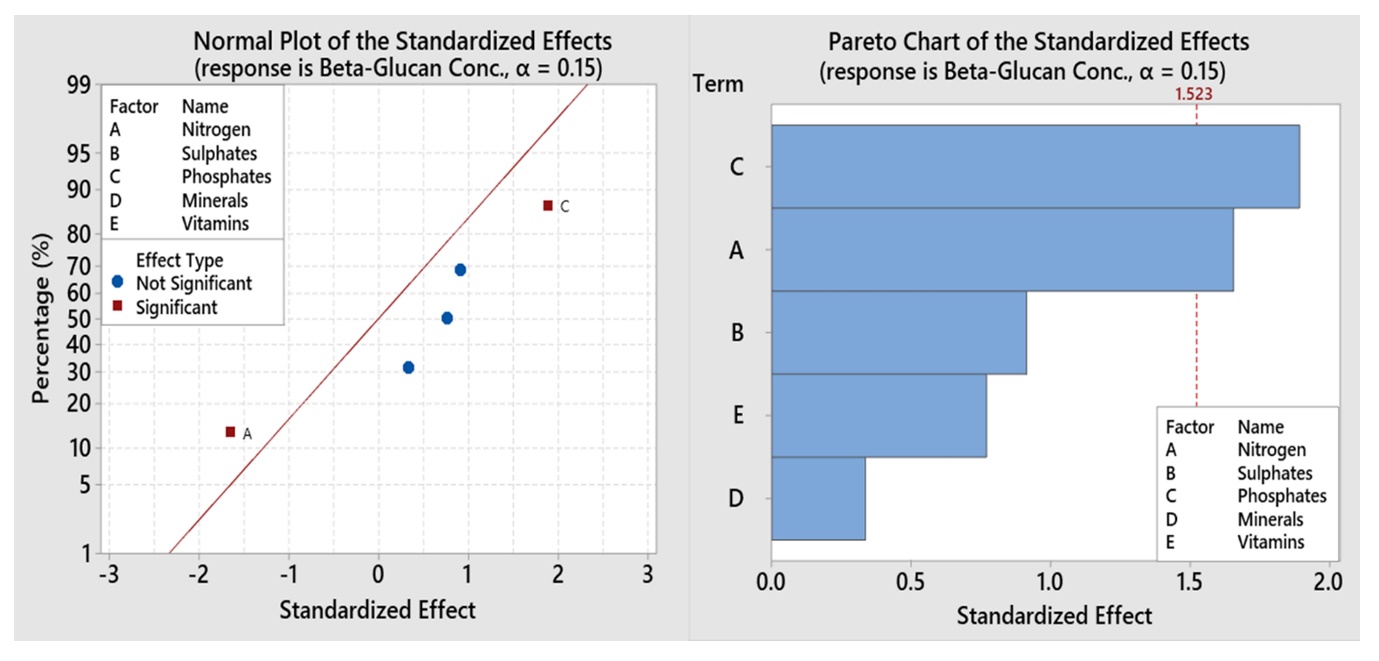
**

**Figure (S2): Plot of significance effects and Pareto chart of the variables influencing β-glucan biosynthesis by *Meyerozyma guilliermondii***

A

B

**Figure (S3): Response optimization plots of the most significant variables affecting β-glucan biosynthesis:**

**A: Minerals and vitamins for *Kluyveromyces lactis***

**B: Nitrogen and Phosphate concentrations for *Meyerozyma guilliermondii***

**
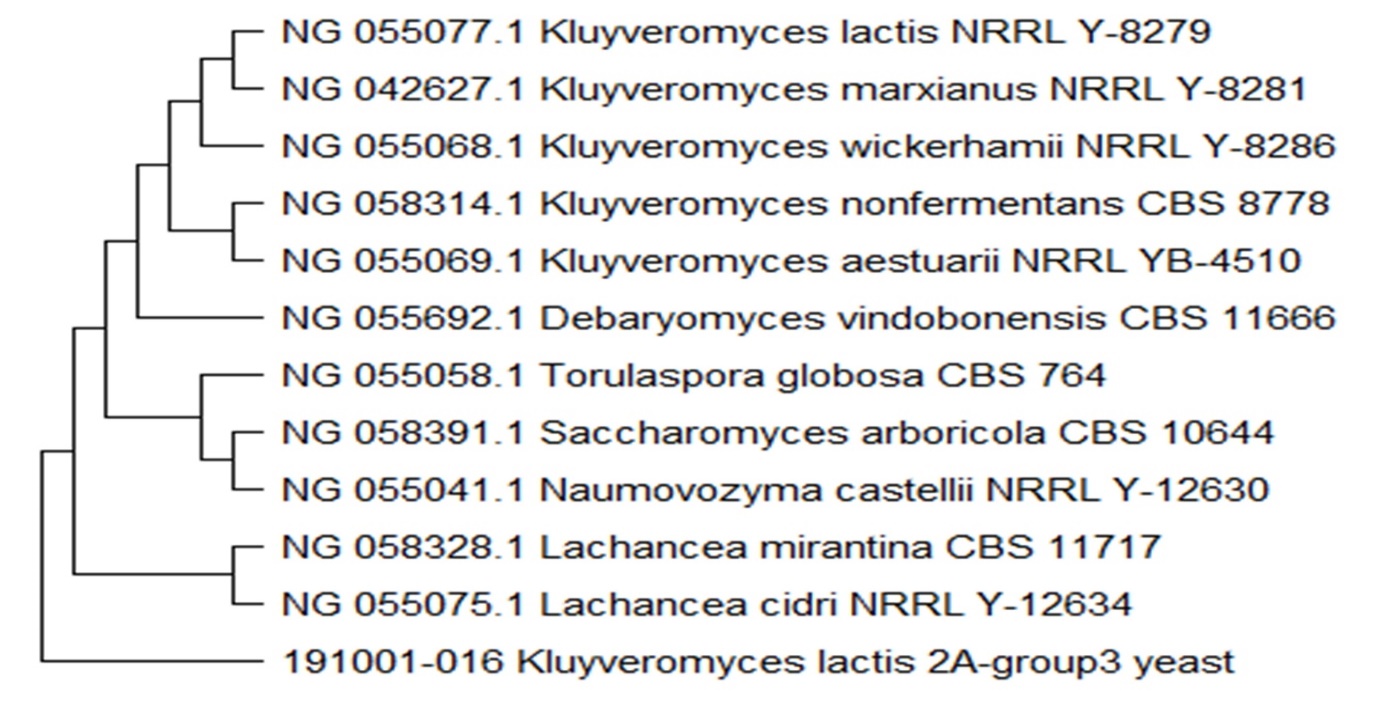
**

**Figure S4. Phylogenetic tree showing the relationship of the selected yeast *Kluyveromyces lactis* with other related fungal species relatives from Genbank based on their sequence homology of 18S rRNA**

**
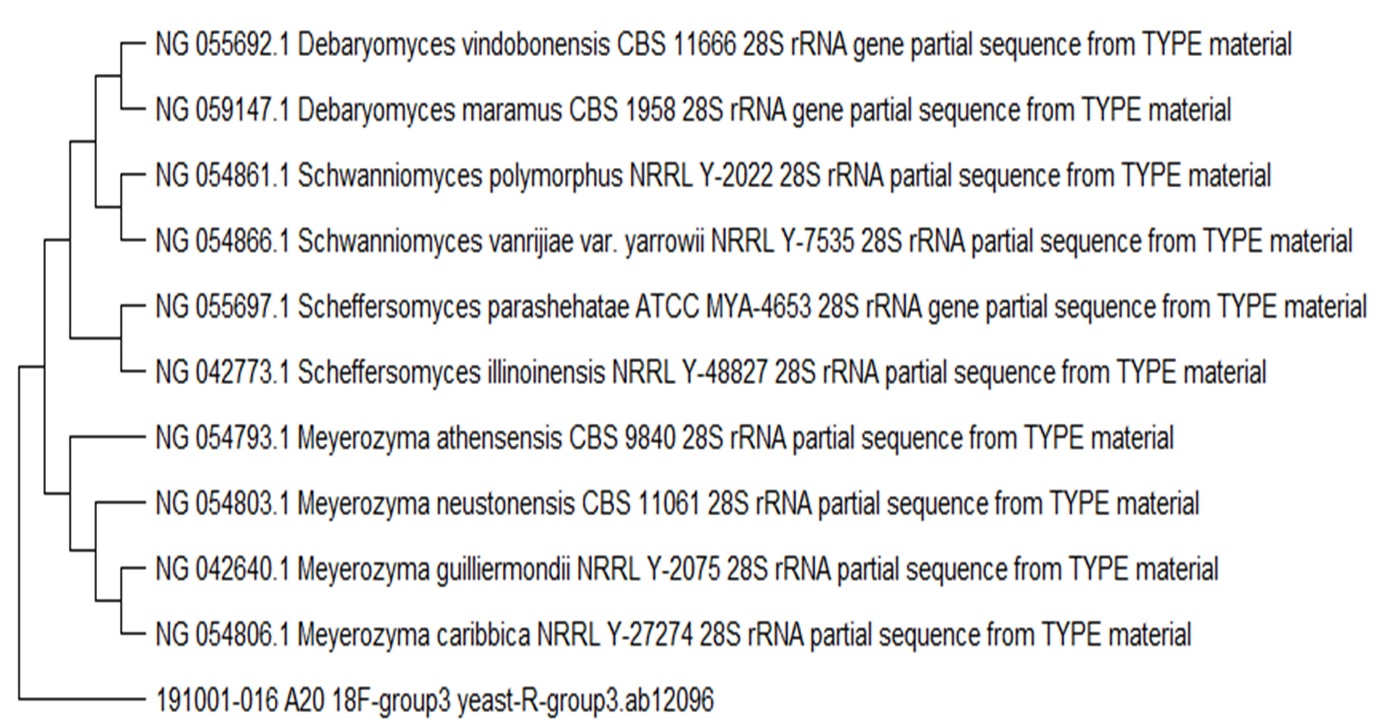
**

**Figure S5. Phylogenetic tree showing the relationship of the selected yeast *Meyerozyma guilliermondii* with other related fungal species relatives from Genbank based on their sequence homology of 18S rRNA**
